# Design and Performance Research of a Precision Micro-Drive Reduction System without Additional Motion

**DOI:** 10.3390/mi13101636

**Published:** 2022-09-29

**Authors:** Manzhi Yang, Xiaodong Zhang, Chuanwei Zhang, Hongzhang Wu, Yizhi Yang

**Affiliations:** 1 College of Mechanical Engineering, Xi’an University of Science and Technology, No. 58 Yanta Middle Road, Xi’an 710054, China; 2 Xi’an Institute of Metrology, Xi’an 710068, China; 3 College of Humanities and Foreign Languages, Xi’an University of Science and Technology, No. 58 Yanta Middle Road, Xi’an 710054, China

**Keywords:** micro-drive system, flexible hinge, PZT, kinematic performance

## Abstract

A micro-drive system is a key part of macro-micro-drive technology and precision positioning technology in which a micro-drive reduction system can provide more precise motion and suitable small space motion. Therefore, it is necessary to study precision micro-drive reduction systems. In this paper, based on the design of a micro-drive reduction mechanism without force and displacement in non-motion direction, a precision micro-drive reduction system driven by a piezoelectric ceramic actuator (PZT) was designed, and the strength, dynamic and motion performance of the system was analyzed. First, based on the principle of a flexure hinge lever and the principle of balanced additional force, a type of precision micro-drive reduction mechanism with an adjustable reduction ratio was designed. Second, the strength performance of the system was analyzed by finite element analysis, and the dynamic performance of the system was analyzed by finite element analysis and experiments. Finally, the kinematic performance of the system was analyzed by theoretical analysis, the finite element method and experiment, and the motion linear equation was calculated based on the linear fitting equations of three methods. The study results showed that the system had good strength and dynamic performances, and the system’s motion had the advantages of high precision and good linearity. This research has certain reference value for the design and performance research of micro-drive mechanisms.

## 1. Introduction

In recent years, micro-drive systems have been widely used in the military and aerospace industries and in biological engineering. How to accurately drive and transform the input displacement has become the focus of micro-drive system research. A micro-drive system can not only be used in macro-micro-drive systems to compensate for the macro-drive error and improve the accuracy of the overall system [1,2,3], but it can also be used independently in various finishing and micro-displacement occasions to produce precise linear displacement and precise positioning. Micro-drive systems play an important role in promoting the development of precision engineering, microelectromechanical system technology, microelectronic technology, nanotechnology, modern biotechnology, and other fields. A micro-drive system based on a micro-drive mechanism can produce tiny and precise linear displacement and is an important feeding and positioning element in precision machining and high-precision instruments [4,5,6,7].

Many scholars have conducted in-depth research on the micro-drive mechanism. Liu Y. et al. designed a new type of large-stroke micro-clamping device based on a flexure hinge structure, and they adopted a two-stage flexible amplifier mechanism design to realize the stroke amplification of a piezoelectric driver with minimal displacement in the nonworking direction, which can be applied to various micro-assembly occasions [8]. Martin L. Culpepper et al. from MIT designed an ultraprecise optical alignment mechanism driven by an electromagnetic force based on a flexible mechanism; when the installation deviation of this mechanism was less than ±1 mm, the overall system error was less than 0.1%, the working stroke of the mechanism was 100 × 100 × 100 nm, and the displacement resolution was less than 5 nm [9]. S. K. Nah and Z. W. Zhong, from Nanyang Technological University, developed a flexible micro-gripper. The micro-gripper was driven by piezoelectricity, which could amplify the displacement by three times and had a maximum motion stroke of 170 µm; the simulation and experimental results proved that the gripper had good performance [10]. Tung-li Wu et al. designed a nanoscale positioning device (6-PSS configure ration) driven by six piezoelectric ceramic actuators mounted directly on the base, which had the advantages of fast response, high stiffness and a small mechanical circuit; the experimental results showed that the device could achieve an 8 μm motion stroke at a resolution of 5 nm, and it can be used to operate equipment with nanoscale motion [11]. The above studies on the motion of micro-drive systems mainly focused on the amplification motion, and there is less research on micro-drive reduction systems than micro-drive insufficient systems [12,13,14,15]. A micro-drive reduction system can improve the motion accuracy, increase the scope of application of the micro-drive and provide suitable small-space motion [16,17,18,19,20,21]. Therefore, it is of great significance to study micro-drive reduction systems.

Feng S. et al. proposed an equal-stiffness and equal-stroke 2D micro-positioning platform which was driven by piezoelectric actuators, where the overall structure of the 2D micro-positioning platform adopted a nested structure and the displacement magnification mechanism adopted two hourglass displacement magnification mechanisms [22]. Xue X. presented the design, fabrication and experimental tests of a piezo-driven positioning stage based on the inchworm principle, which could provide a large range of motion with high resolution and high stiffness [23]. Tanikawa T. et al. designed a thin-plate 3-DOF parallel mechanism for a micro finger module in microoperation. The research showed that the micro hand with two fingers, based on the movement principle of a tweezer, was effective for the operation of micro-objects and could move in a space requiring a small size and high precision [24]. Panin F. et al. described a mechanical component that could produce minimal rotation of its output parts; a very high displacement reduction ratio was achieved entirely through the elastic deformation of the parts, its resolution was one to two orders of magnitude higher than the most advanced devices, there was no backlash friction, and the design was simple and compact [25]. Sakuma S. et al. proposed a reduction mechanism to achieve a nanoscale resolution for the positioning of the tip of a probe. The mechanism used a combination of springs with different levels of stiffness, driven by magnetic forces, to evaluate the performance of the prototype device, and the results showed that the standard deviation of the tip’s displacement was less than 0.18 μm as a measure of repeated positioning accuracy [26]. In the motion process of the micro-drive reduction mechanism, the force and displacement in the non-motion direction usually produce a certain parasitic motion [27,28,29,30]. The strength of the micro-driver in the non-motion direction is generally poor, and the parasitic movement easily damages the micro-driver, affecting the precision and security of the system in the process of movement. Therefore, the elimination of additional motion is of great significance in the study of the micro-drive mechanism [31,32,33,34].

In order to solve the problem of force and displacement in the non-motion direction of micro-drive reduction, this paper designed a micro-drive reduction system without additional force and displacement. In addition, the strength, dynamic and motion performance of the system was analyzed. The results showed that the system had good strength and dynamic performance and met the design requirements, and the precision and linearity of the system motion were high.

The rest of this paper is organized as follows: Section 2 describes the design of the precision micro-drive reduction system and introduces the working principle of the micro-drive reduction mechanism, while the reduction ratio of the micro-drive reduction system is also calculated. In Section 3, the strength, modal and kinematics of the system are analyzed by the finite element method. In Section 4, the performance experiments of the micro-drive reduction system are studied. Finally, the conclusion is provided in Section 5.

## 2. Design of the Precision Micro-Drive Reduction System

### 2.1. System Structure Design

**Figure 1:**
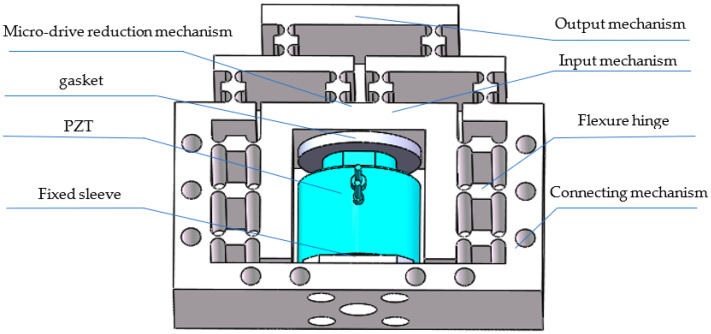
Structure diagram of the micro-drive reduction system.

Figure 1 shows a 3D model of the designed micro-drive reduction system. The reduction ratio of the mechanism can be adjusted according to the actual needs; because 0.5 was convenient for this study, this paper took the reduction ratio of 0.5 as an example for the design. The PZT provides the input displacement of the system. The fixed sleeve and the connecting gasket connect the PZT and the transmission micro mechanism. The micro-drive system is fixed on a working table by the connecting mechanism, and the driving displacement is introduced into the system from the input mechanism. After the displacement is reduced by the design ratio of 2:1, the output mechanism is outputted at the upper end.

### 2.2. Design of the Micro-Drive Reduction Mechanism

**Figure 2:**
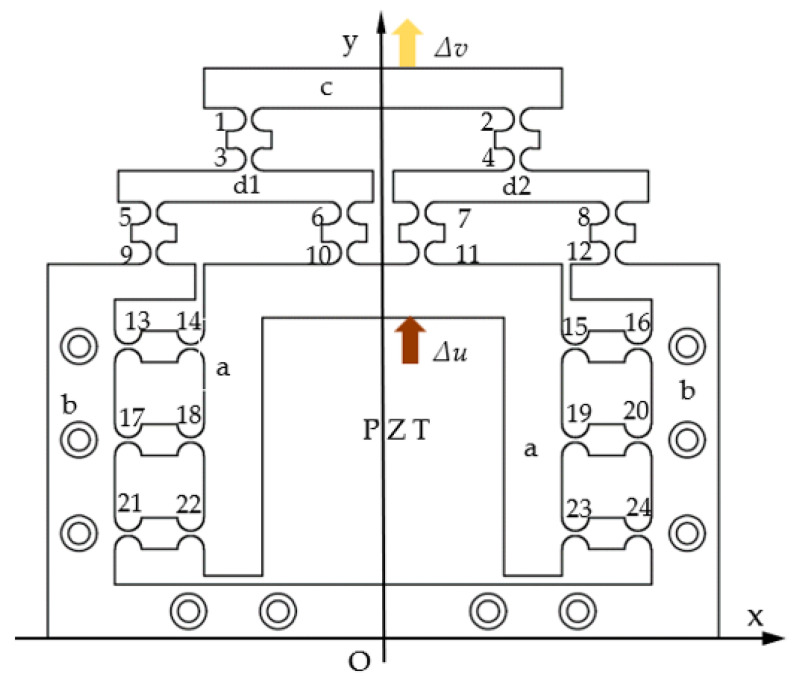
Schematic diagram of the micro-drive reduction mechanism.

According to the principle of the lever and balancing additional force, a micro-drive mechanism was designed that could accurately reduce the movement input. A schematic diagram of the structure is shown in Figure 2, which contains 24 straight-round flexure hinges. The radius of hinges 1–12 was 2.5 mm, and the minimum distance was 1 mm. Hinges 13–24 had a radius of 3 mm and a minimum distance of 1 mm. A PZT was placed in the center to drive the mechanism, and 10 *M4* screws were externally used to fix the mechanism onto the workbench. The mechanism can be designed according to the actual needs, and the size of the reduction ratio can be adjusted. This design was for a specific proportion (i.e., a reduction ratio of 2:1).

The external dimensions of the micro-drive reduction mechanism (i.e., X × Y × Z) were 150 × 128 × 50 mm, and a rectangular coordinate system was established as shown in Figure 2. Under the action of the micro-driver, the input mechanism (a) obtained the motion input (Δ*u*), and under the action of the guiding principle of the flexible hinge, the mechanism guided the motion input forward along the *y*-axis and entered the reduction mechanism (d). Under the action of the lever principle, the input displacement was reduced proportionally, and the motion error was also reduced; that is, the motion precision improved. The high precision motion displacement (Δ*v*) was outputted at the top of the output mechanism (c). In the process of motion, flexible hinges 13–24 were symmetrically distributed on both sides of the piezoelectric ceramic. According to the principle of balancing additional force, the non-motion direction (*x*-direction) forces generated by the mechanism in the process of motion will cancel each other, ensuring the safety of the driver and the precision of the output displacement.

### 2.3. Analysis of the Mechanism’s Working Principle

#### 2.3.1. Principle of Lever

The lever principle can accurately realize a reduction in motion input and other precision displacement changes. The practical application of the lever principle and its correctness and validity were used in this study as a working principle of the micro-drive reduction system, and the design of the micro-drive reduction mechanism for studying concrete was based on the lever principle. In view of the fact that there are many reduction occasions in practical application, a micro-drive reduction mechanism with a precisely adjustable reduction ratio was designed.

**Figure 3:**
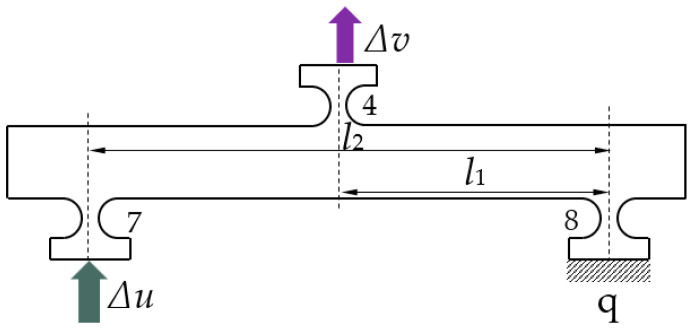
Schematic diagram of the lever working principle.

The working principle of the lever reduction for the micro-drive reduction system was analyzed as shown in Figure 3. The right endpoint q is connected with the experimental platform and was the fixed endpoint. Under the action of the input displacement (Δ*u*), the input end moved along the direction shown in Figure 3, and the end point, q, was fixed. Under the action of the lever principle, the input displacement was reduced by the ratio *l*_1_*/l*_2_, and the output end moved along the direction shown in Figure 3, and the output displacement Δ*v*.

#### 2.3.2. Principle of a Balancing Additional Force

**Figure 4:**
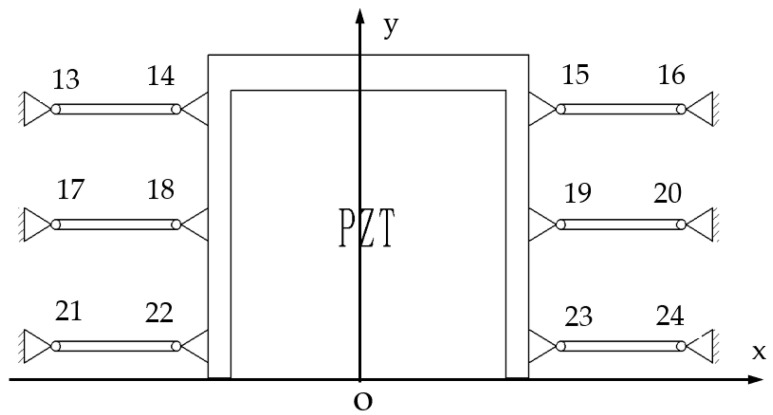
Principle diagram of the balanced additional force.

The principle of a balancing additional force for the micro-drive reduction mechanism is shown in Figure 4. In the motion process of the micro-drive reduction mechanism, additional forces in the non-motion direction (*x* direction) are generated, the PZT is brittle in the *x*-direction, and it is easily damaged by force. Therefore, six flexure hinge components symmetrically distributed on the *y* axis were designed to balance the additional forces in the non-motion direction.

In the process of motion, the additional force generated in the *x*-direction has the same size and opposite direction, which cancel each other out, realizing a balance of the additional force of the micro-drive mechanism, preventing the existence of a transverse additional force in the process of motion, thus ensuring the safety of the PZT in the process of motion.

At the same time, when the system moves in the x direction, its displacement will cancel with the tiny deformation of the flexible hinge 13–24, so it can only be generated in the direction of motion. This ensures the precision of the motion of the system.

Therefore, in the process of system movement, it has no non-motion direction force and displacement, to ensure the safety and precision of the system.

#### 2.3.3. Working Principle of the Mechanism

Based on the equivalent simplified diagram of the flexure hinge component in Figure 2, the flexure hinge in the micro-drive reduction mechanism was simplified, and the flexure hinge component was equivalent to a rod. A diagram of its working principle is shown in Figure 2. The input displacement of the micro-drive reduction system was Δ*u*, and the output displacement was Δ*v*. The fulcrum of the flexible hinge 9, 12, 13, 16, 17, 20, 21, and 24 was connected with connecting mechanism b, and the whole micro-drive system was fixed on the workbench through connecting mechanism b. The fulcrum of the flexure hinges 10, 11, 14, 15, 18, 19, 22, and 23 was connected with the input transmission mechanism a. The fulcrum of the flexible hinges 3, 4, 5, 6, 7 and 8 was connected with mechanism a (the symmetric structure includes d1 and d2). The fulcrum of the flexible hinge 1 and 2 was connected with output mechanism c, and the output displacement of the micro-drive system was outputted by output mechanism c. The flexible hinges 13–24 guided the linear motion of the driver and balanced the forces generated in the non-motion direction during movement. The main function of the micro-drive reduction system was to make use of the guiding function of the flexible hinge mechanism and the principle of lever reduction to precisely reduce the input linear displacement and to transfer and output the linear displacement.

### 2.4. Reduction Ratio Calculation

**Figure 5:**
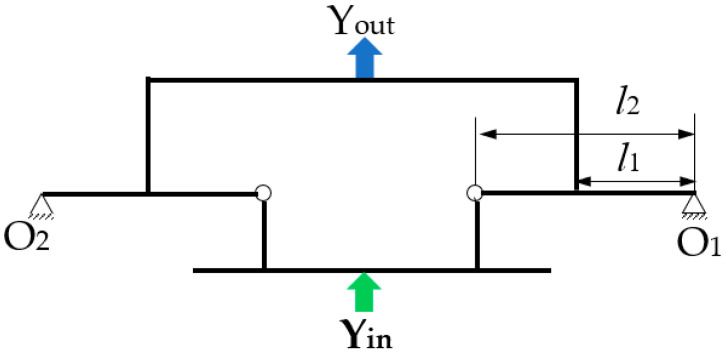
Principle diagram of micro-drive reduction mechanism.

A 3D model of the reduction micro-drive system is shown in Figure 1, and the reduction principle of the micro-drive mechanism is shown in Figure 5. The reduction ratio of the designed precision micro-drive reduction mechanism can be adjusted according to the actual requirements; specifically, the length of the lever between the flexure hinges in the reduction mechanism can be adjusted, that is, the length of *l_i_* in Figure 5. When calculating the reduction ratio, the right half of mechanism (the lever composed of flexible hinges 7, 4, and 8) is used as an example, and its structure can be seen in Figure 3.

**Figure 6:**
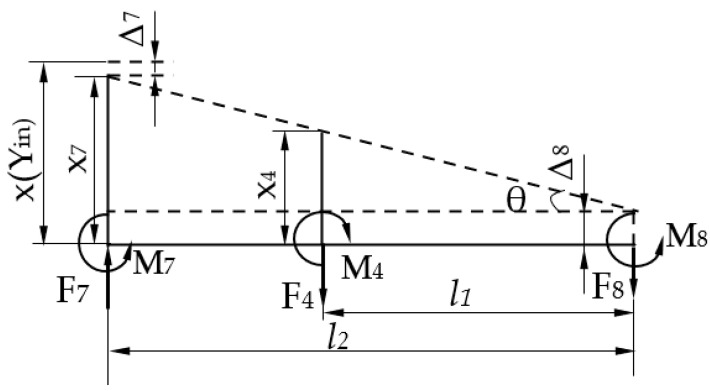
The geometry model of the mechanism with reduction ratio calculation.

When the micro-drive mechanism moves, its reduction ratio will change due to the deflection, stretching and compression of the flexible hinges; the geometry model is shown in Figure 6. Suppose the axial force of flexure hinge *i* (*i* = 1–24) is *F_i_*, the torque is *M_i_*, the axial deformation produced by flexure hinge *i* is *∆_i_*, and the rotation angle is *α_i_*, then the relation between the amount of deformation and the force and torque can be expressed as follows;

(1) $$F_{i}{=K}_{F}\Delta_{i}\left(i=4,7,8\right)$$

(2) $$M_{i}{=K}_{M}\alpha_{i}\left(i=4,7,8\right)$$

among them;

(3) $$K_{F}=\mathrm{Eb}(\frac{2\left(2s+1\right)}{\sqrt{4s+1}}\arctan\sqrt{4s+1}\;-\;\frac{\pi }{2}{)}^{-1}$$

(4) $$K_{M\;}=\frac{\mathrm{EbR}}{12}\;(\frac{{2s}^{3}{(6s}^{2}+4s+1)}{{\left(2s+1)(4s+1\right)}^{2}}+\frac{{12s}^{4}\left(2s+1\right)}{{\left(4s+1\right)}^{\frac{5}{2}}}\arctan\sqrt{4s+1}{)}^{-1}$$

In Equations (1)–(4),

*K_F_* represents the axial tension-compression stiffness of the flexure hinge;
*K_M_* represents the angular stiffness of the flexure hinge;
*E* represents the elastic modulus of the mechanism material;
*s* represents the ratio of the cutting radius of the flexure hinge to the minimum thickness.

For the convenience of analysis, the action force and reaction force of the flexible hinge and its connecting part are represented by the same symbols. Analyze the force and input and output displacement of the lever, as shown in Figure 6.

Suppose that the angle of the lever in the micro-drive mechanism is *θ*, then the relationship between the angle of the flexure hinge α*_i_* and the angle of the rod *θ* is as follows;

(5) $$\alpha_{7}\;=\;\alpha_{8}=\theta$$

According to the force and moment balance, the expression;

(6) $$F_{7}={\;F}_{4}{+F}_{8}$$

(7) $$F_{4}l_{1}{+M}_{7\;}{+M}_{8}={\;F}_{7}l_{2\;}{+M}_{4}$$

The lever takes hinge 8 as the rotation center. Hinge 8 is subjected to the tensile force *F*_8_, and the axial elongation is Δ_8_. Under the action of the piezoelectric ceramic driven force, hinge 7 is axially compressed, and the compression amount Δ_7_. *x*_4_ is the output displacement of the output end of the lever; set *x* as the driven displacement Y_in_ of the piezoelectric ceramic under the load condition, then the input displacement *x*_7_ of the input end of the lever was;

(8) $$x_{7}=x-{\;\Delta }_{7}$$

Then the rotation angle *θ* of the lever was;

(9) $$\theta =\frac{x_{7}-\Delta_{8}}{l_{2}}=\frac{x_{4\;}-\Delta_{8}}{l_{1}}$$

Then,

(10) $$\lambda \;=\frac{x_{4}}{x_{7}}=\frac{\theta l_{1}+\Delta_{8}}{\theta l_{2}+{\;\Delta }_{8}}$$

Combining the above Equations (1)–(10), and substituting *l*_1_ = 15 mm, *l*_2_ = 30 mm, *t* = 1 mm, *R* = 3.6 mm, *b* = 10 mm, and *E* = 206 MPa into the formula to calculate. The reduction ratio of the above the micro-drive reduction mechanism is λ = 0.5.

It can be seen from the analysis and calculation results that the reduction ratio of the micro-drive reduction mechanism designed in this paper is accurate, and the reduction ratio can be adjusted by adjusting the length of the key rod according to the use needs.

### 2.5. Micro-Driver Selection

**Figure 7:**
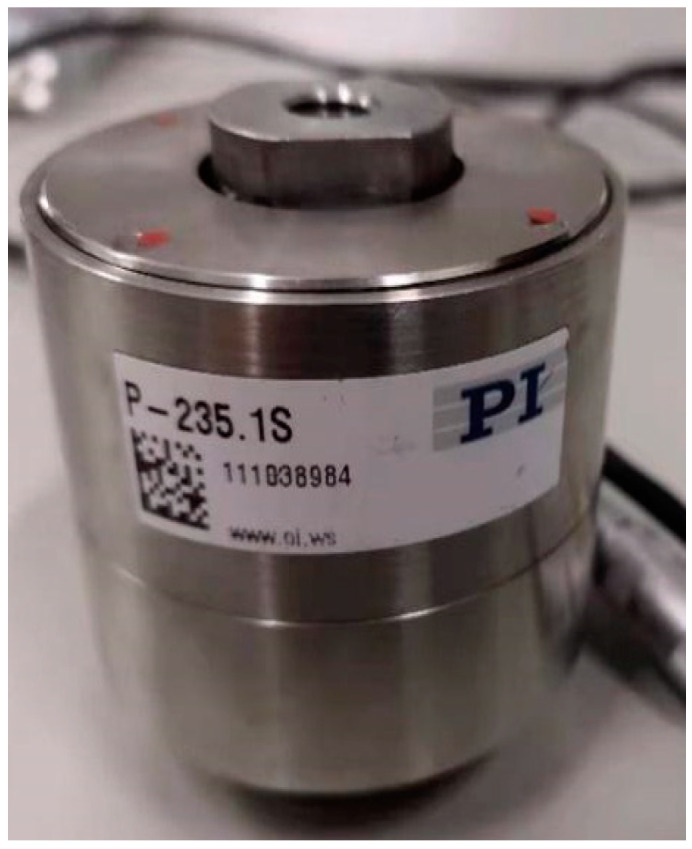
The P-235.1s PZT.

According to the requirements of the output force and the output displacement of the micro-drive system, P-235.1s PZT, made by the PI company in Karlsruhe Germany, was selected for comparing the current use of the micro-positioning actuators. The maximum elongation of piezoelectric ceramics was 15 μm, and the maximum motion frequency was 300 Hz. The parameters are shown in Table 1. PZT is shown in Figure 7.

The P-235.1s PZT selected in this paper could realize closed-loop control of the micro-drive through its own built-in capacitance sensor as a feedback element. The closed-loop control method can reduce the influence of hysteresis, creep and nonlinearity on the driving precision of the PZT.

## 3. Performance Analysis of the Reduction System

**Figure 8:**
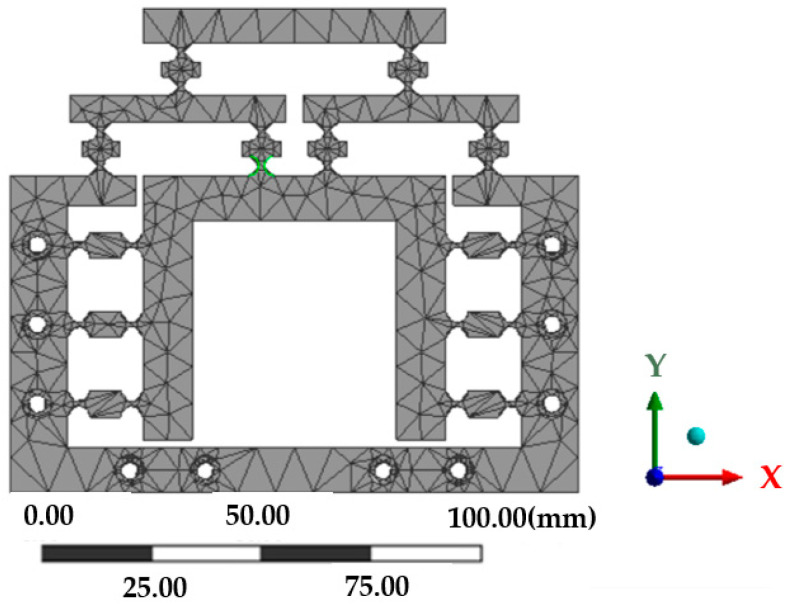
Meshing of the micro-drive reduction mechanism.

In order to analyze the performance of the micro-drive reduction system, the finite element method was used to analyze the strength, modal and kinematic properties of the system, and the 60Si2Mn parameters were selected for the material properties. The simplified 3D model of the micro-drive reduction mechanism was imported into the finite element model. When meshing, the whole model was firstly divided into 1 mm grids as a whole, and then the mesh cells of the 24 cylindrical surfaces with 12 single-chain guides were refined with a parameter refinement of 0.5 mm. The finite element meshed model is shown in Figure 8. It can be seen in Figure 8 that the mesh division of the key parts was fine and smooth without a crossed or fractured network, and the mesh division was of good quality. The model was divided into 203,05 units and 10,913 nodes.

### 3.1. Strength Analysis

**Figure 9:**
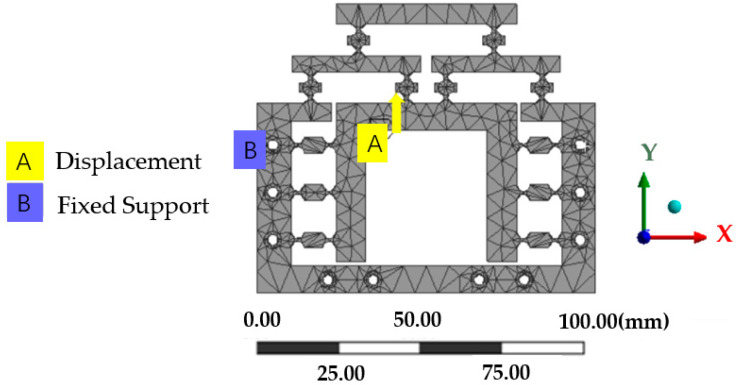
Fixed constraints and applied positive displacement of the *y*-axis.

**Figure 10:**
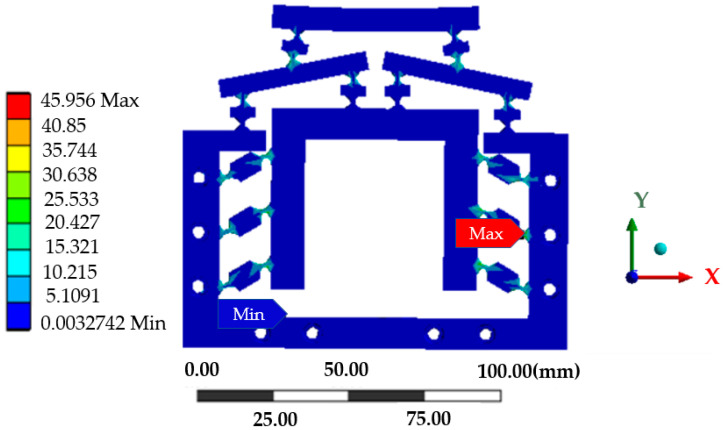
Stress cloud of the micro-drive mechanism.

The static module of the finite element software ANASY2020 R2 was used to analyze the mechanism, and the contact surface between the model and the piezoelectric ceramic driver was fixed to generate two surface marks with the same shape as the contact surface of the piezoelectric ceramic driver to realize the loading. A fixed constraint was applied to the 10 bolt holes on the micro-drive reduction mechanism, and a positive displacement of the *y*-axis was applied at the position of the actuator as shown in Figure 9. A maximum forward displacement of the *y*-axis of 15 μm was applied as a solution. The obtained stress cloud map is shown in Figure 10. The maximum simulated stress of the micro-drive reduction mechanism is 45.956 MPa.

The allowable stress of the material [σ]  was;

(11) $$\left[\sigma \right]=\frac{\sigma_{s}}{n}$$

By putting on 60Si2Mn a yield limit of $\sigma_{s}$ = 1176 MPa and setting a safety factor of *n* = 1.5 into Equation (11), the allowable stress of the material [σ] is 784 MPa, while the maximum simulated stress of the mechanism was only 45.956 MPa, which was far less than the allowable stress for 60Si2Mn.

Therefore, the strength of the micro-drive reduction system met the design requirements and had good strength performance.

### 3.2. Modal Analysis

In order to analyze whether the micro-drive reduction mechanism would produce resonance in the process of motion and cause damage to the system, it is necessary to conduct free mode analysis on the micro-drive reduction mechanism. The preliminary processing of finite element analysis was similar to that of the strength analysis.

**Figure 11:**
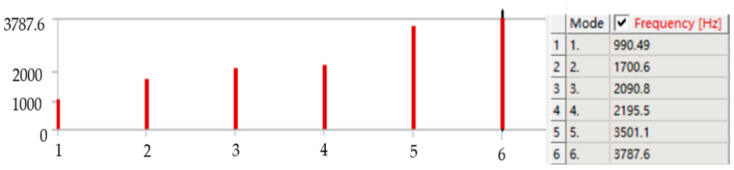
The first six natural frequencies of the micro-drive mechanism.

The first six order natural frequencies of the micro-drive reduction mechanism are shown in Figure 11. According to the analysis results, the first six order natural frequencies were 990.49, 1700.6, 2090.8, 2195.5, 3501.1 and 3787.6 Hz.

### 3.3. Kinematic Analysis

**Figure 12:**
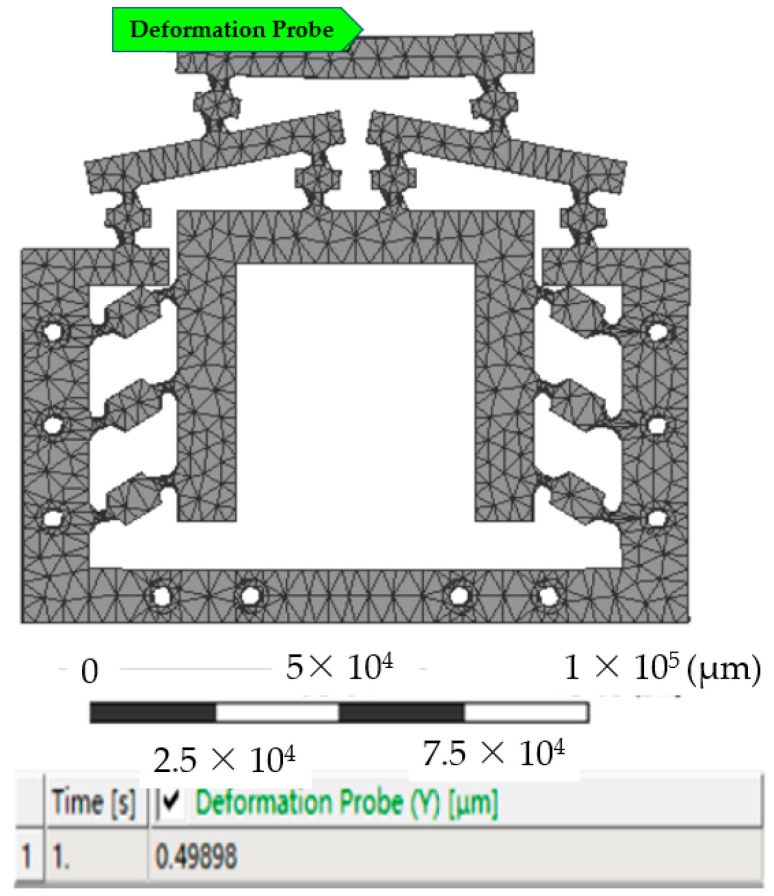
Kinematic analysis of the input displacement at 1 μm.

When the system inputs a linear displacement Δ*u* in the range of 0–7 μm, the displacement output Δ*v* of the micro-drive reduction mechanism was analyzed. The preliminary finite element treatment and constraint conditions were similar to the strength analysis. When the input displacement was 1 μm in the finite element software, the results of the kinematic analysis diagram of the system completed by the probe function was shown in Figure 12. At this time, the system’s Y displacement was 0.49898 μm. According to the same calculation method, the kinematic simulation results of the micro-drive reduction system at 0–7 μm are shown in Figure of 4.2 Results of the analysis of motion performance of the miniaturized system..

## 4. Experiment and Analysis

**Figure 13:**
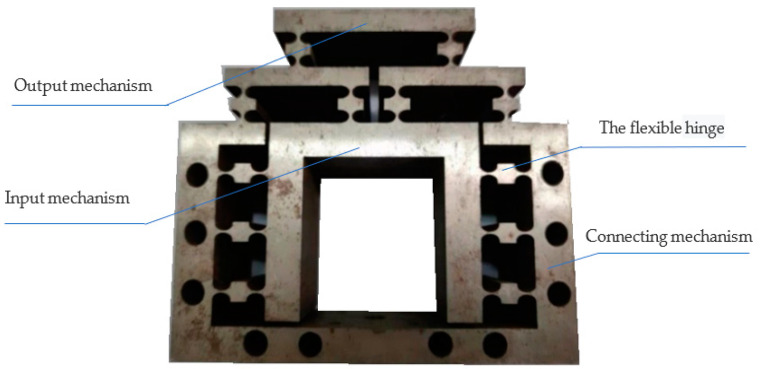
Physical picture of the micro-drive reduction mechanism.

The physical figure of the micro-drive reduction mechanism after processing is shown in Figure 13. The dynamic performance experiment and the performance experiment of the micro-drive reduction system were carried out by using the micro-drive mechanism.

### 4.1. Dynamic Performance Experiment

**Figure 14:**
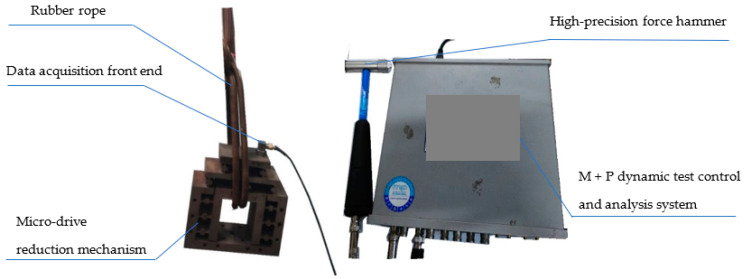
Diagram of the experimental dynamic performance of the micro-drive reduction mechanism.

**Figure 15:**
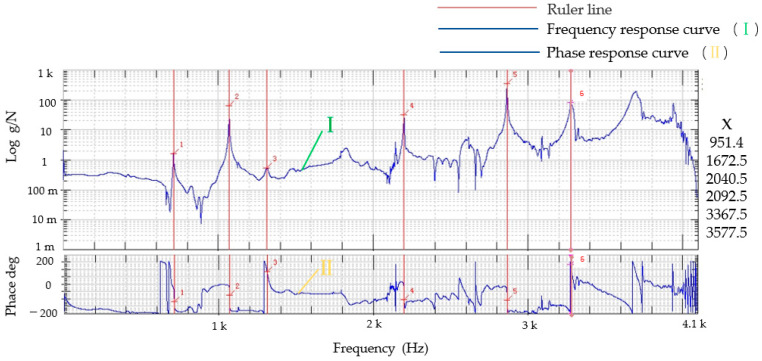
Experimental results of the dynamic performance of the micro-drive reduction mechanism.

The dynamic performance experiment equipment included an M + P dynamic test control and analysis system, a data acquisition front end, a high-precision force hammer, a rubber rope, a micro-drive reduction mechanism, and a computer. The free dynamic experiment was carried out on the micro-drive reduction mechanism. The mechanism was suspended freely with a rubber rope, and the data acquisition front end was fixed on the mechanism. The other end of the data acquisition line was connected with the M + P dynamic test control and analysis system. The dynamic performance experiment on the micro-drive reduction mechanism is shown in Figure 14, and the experimental results are shown in Figure 15.

The first six order natural frequencies of the micro-drive reduction mechanism were analyzed by the finite element method and experiment, and the results are shown in Table 2.

According to the simulation analysis and experimental results of dynamic performance, the following results were obtained:

(1) The finite element analysis and experimental analysis results were relatively consistent, and the maximum error was 6.04%, indicating that the analysis results were accurate and reliable, and the lowest natural frequency was approximately 990 Hz, which had good dynamic performance.
(2) In the micro-drive reduction system, P235.1s PZT was used to drive, and its maximum frequency was 300 Hz, which did not resonate with the micro-drive reduction mechanism; thus, the micro-drive reduction system had a good dynamic performance.

Therefore, the micro-drive reduction system had a good dynamic performance and met the design and application requirements.

### 4.2. Reduction Performance Experiment

The design reduction ratio of the micro-drive reduction system was 2:1. The finite element analysis method was used to analyze its reduction performance, and the experimental method was used to verify its reduction performance. In order to verify whether the reduction ratio was accurate, the value was verified within the input range of 0–7 μm.

**Figure 16:**
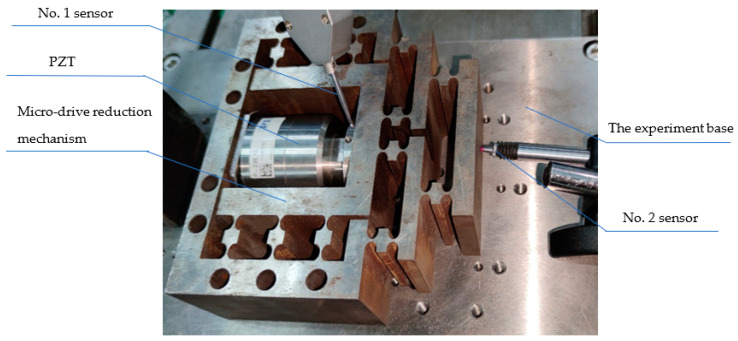
Diagram of the experiment on the micro-drive reduction system.

**Figure 17:**
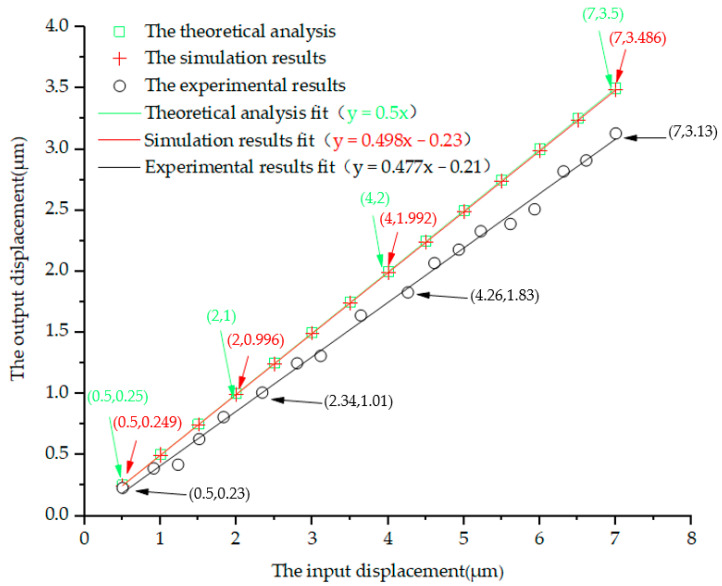
Results of the analysis of motion performance of the miniaturized system.

The reduction performance experiment’s equipment included the P-235.1s PZT; two inductive sensors (i.e., the No. 1 sensor was a side type, and its resolution was 0.03 μm; the No. 2 sensor was a straight sensor, and its resolution was 0.05 μm); the micro-drive reduction mechanism; an experimental base; an MDG-8 data acquisition. The reduction performance experiment, as shown in Figure 16, was equipped with a micro-drive reduction mechanism with the PZT in the experiment with the base, using the No. 1 sensor as the input displacement measuring system and the No. 2 sensor as the output displacement measurement system; the experimental result as shown in Figure 17.

The theoretical analysis, simulation results and experimental results within the range of 0–7 μm of the input displacement were fitted, as shown in Figure 17.

The linear Equation obtained by fitting the theoretical analysis values was;

Δ*v* = 0.5Δ*u* (12)

Its linearity was 1;

The linear Equation obtained by fitting the finite element analysis values was;

Δ*v* = 0.498Δ*u* − 0.23 (13)

Its linearity was 1;

The linear Equation obtained by fitting the finite element analysis values was;

Δ*v* = 0.477Δ*u* − 0.21 (14)

Its linearity is 0.994.

It can be seen from the fitted linear Equations (12)–(14) that the input displacement Δ*u* and output displacement Δ*v* of the micro-drive reduction system had high linearity. The error between the theoretical analysis value and experimental value calculated by using the slope error of the fitted linear equation is 4.82%, and the error between the finite element analysis value and experimental value is 4.4%. Thus, the precision of the system output was high.

In order to facilitate practical application, the linear Equations (12)–(14) fitted above can be approximately described by the linear Equation (15) combined with the characteristics of the micro-drive reduction system;

Δ*v* = 0.492Δ*u* (15)

The linear Equation (15) is the motion linear equation of the system.

According to the result of the analysis of the kinematic performance, the micro-drive reduction system between the theoretical analysis value and experimental value was 4.82%, and the maximum error was 0.37 μm. The error between the finite element analysis value and experimental value was 4.4%, and the maximum error was 0.356 μm. Therefore, the micro-drive reduction system has better motion performance and higher positioning accuracy.

## 5. Conclusions

In this paper, a precision micro-drive reduction system without additional force and displacement was designed, and the strength, dynamic and motion performance of the system were analyzed. The simulation and experimental results showed that the system had good strength performance and dynamic performance, and the system had the advantages of high precision and good linearity during its motion.

A precise micro-drive reduction system with an adjustable reduction ratio was designed, and it has no non-motion direction force and displacement during motion because of its own structure, so as to ensure the safety and precision of the system. The strength analysis and dynamic performance analysis of the system are completed by finite element analysis and experiment.

The results of the analysis of the motion performance showed that the system had high positioning accuracy and good motion linearity, the maximum motion error of the system was 4.82%, the maximum error was 0.37 μm, and the minimum linearity of the system was 0.994. Future work should focus on optimizing the structure and improving the accuracy of the system.

## Figures and Tables

**Table 1:** Main technical parameters of the P-235.1s PZT [35].

Indicators	Parameter
The length of the PZT	55 mm
The closed loop displacement	0–15 μm
The resolution of the	0.3 nm
Maximum frequency of motion	300 Hz
Maximum thrust	30,000 N
Maximum tension	3500 N
Maximum shear force	707 N
Maximum bearing moment	2 Nm
Working voltage	0–10 V

**Table 2:** Comparison between the finite element method and experimental dynamic analysis.

Order Time	Finite Element Calculation of Natural Frequency Values (Hz)	Experimental Natural Frequency Value (Hz)	The Relative Error (%)
1	990.49	951.4	4.11
2	1700.6	1672.5	1.68
3	2090.8	2040.5	2.47
4	2195.5	2092.5	4.92
5	3501.1.	3367.5	3.97
6	3787.6	3577.5	6.04

## Data Availability

The data presented in this study are available on request from the corresponding author.
